# To the Profession

**Published:** 1884-10

**Authors:** 


### EDITORIAL DEPARTMENT.

### TO THE PROFESSION.

With this issue of the JOURNAL we close our first year as one of its editors. We have diligently and honestly striven to be true to the very best interests of our profession. We do not feel that we have been able to please all of our readers ; for one who is a radical of radicals, and an ultra among members of the ultra-scientific school, cannot at all times please every one, no matter how true his endeavors may be or have been. We do not, however, write so much to please as to instruct, and we have to thank many of our colleagues for their earnest letters of sympathetic support.

Some of them have said that "they feared we would weary in our undertaking, and that our enthusiasm would burn out." To such we would answer that this journal has mast-headed the flag "Excelsior," and that we intend to lead the advance guard of Veterinary Progress in the United States, not only until we have a well-organized Veterinary Police System in the land, but a National Veterinary Institute, which shall be as fully devoted to Scientific Research as to teaching the principles of Science and Practice to students.

Our endeavors are so well expressed in two resolutions passed at the Brussels Veterinary Congress, that we feel justified in introducing them here, particularly as they should form the professional endeavor of every graduated veterinarian :

"1. A veterinary police and hygienic organization must be inaugurated by every civilized country, which should be intrusted with all the responsibilities that these words imply. Its members must be approved, i. e., graduated, veterinarians from schools of recognized standing ; they should be the counsellors of the civil officials upon all technical questions, and the executors of the laws. A veterinarian should also be appointed as technical adviser to the State Governments as well as to the Central Government, who should be known as the Veterinary-in-Chief of the country.

"2 Such a veterinary police service should seek to employ the largest possible number of veterinarians, in order that the work may be thoroughly done. To such a service belongs the control of all animal markets, meat and animal inspection at abattoirs and knacker establishments, the inspection of fairs, breeding establishments and dairies, and the collection of animal statistics. Further, such a service has to watch over and control the extension of contagious and infectious animal diseases, as well as all similar duties which the interests of the State may require."

We may congratulate the profession on a step in the right direction that has been taken by Congress, authorizing the formation of a "Bureau of Animal Industry," to which we alluded in our last issue. While the authority given to the Commissioner of Agriculture permits the employment of twenty veterinarians, it errs in the first place in not requiring them to be "approved" men,—we can trust the present Commissioner on this point, however,—but errs still more in fixing the pay of such men at the niggardly price of *ten dollars per day.*

The "dead beat" principle, which so often disgraces our public authorities, by which men are expected to serve for "honor" only, or for a price for which none but wealthy or incompetent persons can be had, should be abandoned. In everything "the laborer is worthy of his hire," and never more so than where technical ability is required, which demands not only especial study and fitness, but frequently the neglect of one's business, and in many cases, as in investigations upon disease or its causes, the imperilling of one's life.

In this regard, our Government could well follow the example of that of Germany in its treatment of the Cholera Commission which it lately sent to the East to investigate into the nature and cause of that disease.

Here we find no ten-dollar-a-day business ; but on the contrary, a noble appreciation of the value of the scientific laborer. The Commission, on its return, was given a grand reception, its members honored with decorations, and not only were they well paid when absent, but had any of them died, their families would have been provided for by a liberal pension. They also *presented to the members of the Commission* $33,750, *as an additional reward for their services.*

Under such circumstances, it is no wonder that Science flourishes in Germany, and that men can be found willing to devote their lives to the benefit of the world ; for Germany is not the only country that profits by such researches. The world is the richer, and American life preserved, by the knowledge thus gained, though we are so penurious a people as to be unwilling to do our part in this grand work for the prevention of disease.

During the past year we have felt it our duty to place most energetically before our readers, the injurious influence upon our professional unity and advancement which is sure to result from the example set by the "Subscription Plan," upon which Harvard Veterinary School is conducted. We make no apologies for so often referring to this disagreeable question. We should have been false to every obligation we assumed when we entered the profession, and especially when we assumed editorial responsibilities, had we not done so. We shall continue the fight until right prevails over might, and the governors of this school consent to conduct it upon ethical principles.

In this regard we would say that we sincerely wish that the numerous gentlemen that have privately written us their approval of our action, would be equally explicit in public, either in our own columns or those of some other journal.

During the past year we have had to record unfortunate mistakes made with reference to the "Foot and Mouth Disease," which can have but served to lower our profession in the minds of people both at home and abroad. We may, however, congratulate ourselves that one of them was not made by a professional, but by an empiric, though he has the endorsement of our Government that he is a veterinarian, and possesses abilities which his very actions show he does not possess.

The other mistake was equally serious in the alarm it created, yet we have seen in both instances that the makers of these errors have in the one case been retained in the Government employ, and in the other rewarded by an appointment as State Veterinarian. Surely the ways of our authorities are as incomprehensible in matters veterinary, as upon the majority of questions of public importance.

We have, again, genuine cause for congratulation, in that we have now open one well organized veterinary school that is neither a private endeavor nor run upon a "subscription" basis—one that promises to be at least an able teaching school. We allude to Philadelphia. We hope that another year will see its faculty more replete with veterinarians, and that its work will show that its members have ability for research as well as teaching.

We are pleased to note the formation of several new Veterinary Associations in different States during the past year, and that they have been formed upon a more exclusive basis than many of their predecessors. We wish them success, and would inform them that we should be pleased to publish the reports of their proceedings, as well as such papers as may be considered worthy of publication.

We want assistance. We think we deserve it. We much desire reports, especially statistical, of the frequency with which our colleagues meet with contagious and infectious diseases in their practice. We would call the attention of our readers to a paper of this nature which appears in the present issue, and request that they, one and all, endeavor to do likewise during the course of the coming year. B.

